# The potential role of the lung–brain axis in the pathophysiology of epilepsy: A hypothesis-driven perspective

**DOI:** 10.1016/j.gendis.2026.102240

**Published:** 2026-05-14

**Authors:** Zijun Lin, Di Zhou, Jialing Jiang, Patrick Kwan, Xin Tian

**Affiliations:** a Department of Geriatrics, Laboratory of Research and Translation for Geriatric Diseases, The First Affiliated Hospital of Chongqing Medical University, Chongqing 400016, China; b Department of Neurology, The First Affiliated Hospital of Chongqing Medical University, Chongqing Key Laboratory of Major Neurological and Mental Disorders, Chongqing 400016, China; c Department of Epilepsy Center, The First Affiliated Hospital of Chongqing Medical University, Chongqing 400016, China; d Key Laboratory of Major Brain Disease and Aging Research (Ministry of Education), Chongqing Medical University, Chongqing 400016, China; e CQMU-University of Leicester Joint Institute, Chongqing Medical University, Chongqing 400016, China; f Department of Neuroscience, School of Translational Medicine, Monash University, Melbourne 3004, Australia

**Keywords:** Air pollution, Epilepsy, Lung‒brain axis, Microbiome, Pathogenesis

## Abstract

The pathophysiology of epilepsy remains poorly understood. One of the less explored areas is the role of the lung–brain axis, a sophisticated and intricate bidirectional connection between these two vital organs. Inhaled air pollutants can disrupt lung microbiome homeostasis. This disruption, analogous to gut dysbiosis implicated in neurological conditions, may contribute to epilepsy pathogenesis. Here, we review the existing evidence and theoretical foundations supporting the hypothesis that dysbiosis within the lung microbiota may play a role in the pathophysiology of epilepsy. This includes the links between environmental factors (particularly air pollution) and epilepsy susceptibility; the associations between lung-intrinsic microbiota dysregulation and neurological dysfunction; and the underlying molecular, immunological, and neural mechanisms that enable the lung–brain axis to modulate epileptogenesis. Furthermore, we outline the possible potential pathogenic mechanisms of epilepsy from the perspective of the microbiota–lung–brain axis, offer fresh perspectives on the pathophysiology of epilepsy, and explore potential new research directions related to the lung–brain axis and epilepsy. We propose that a deeper understanding of the function of the lung–brain axis will provide new insights into the etiology, diagnosis, prognosis, and treatment of epilepsy.

## Introduction

The rapid urbanization and industrialization over the past two centuries have spurred societal advancement but have concurrently introduced health risks. Environmental pollutants (*e.g.*, gas emissions, biochemical contaminants, and heavy metals) enter the body via dietary consumption, inhalation, and absorption through the skin; accumulate in the stomach, lungs, and central nervous system (CNS)^1^; and disrupt cellular functions, causing neurotoxicity and immunological dysregulation.^2^ Accumulating evidence suggests that inhalation of air pollutants may predispose individuals to neurological disorders, including epilepsy, through the microbiota-mediated lung–brain axis.^3^^,^^4^ This axis links pulmonary exposure to systemic inflammation, blood–brain barrier disruption, and neuroinflammation, potentially exacerbating epileptogenesis via cytokine signaling (*e.g.*, IL-1β) and microglial activation.^4^

Epilepsy is a prevalent, severe, long-term CNS disorder. As defined by the International League Against Epilepsy (ILAE),^5^ an epileptic seizure is “the transient occurrence of signs or symptoms resulting from abnormal, excessive, or synchronous neuronal activity in the brain”. Recurrent seizures are associated with an increased risk of injury and death. Therefore, seizure control is the primary goal of epilepsy treatment. The mainstay of treatment for epilepsy is antiseizure medications, which are “symptomatic” treatments that aim to suppress seizure occurrence without modifying the underlying disease process. Despite treatment with antiseizure medications, seizures persist in approximately one-third of patients.^6^ This highlights the need to better understand the pathogenesis of epilepsy and develop novel therapies. Given that the cause of epilepsy is unknown in 44%–69% of individuals, there are likely many unrecognized pathomechanisms for epilepsy.^7^ One of the less explored areas is the role of the lung–brain axis. Recent research has reported that higher air pollution levels are associated with an increased risk of both new-onset clinical seizures and subclinical seizures in people with epilepsy,^8^ suggesting that environmental materials may be avoidable seizure triggers. If air pollution increases the risk of epilepsy, reducing exposure might decrease the risk of epilepsy and its associated health consequences.

One mechanism through which air pollution enhances seizure susceptibility may involve the perturbation of the lung microbiota.^9^ The acquired lung microbiota modulates local immunological and neuronal microenvironments and regulates pulmonary inflammation via multiple pathways^10^; moreover, experimental evidence has confirmed that exposure to diverse environmental contaminants can disrupt host homeostasis by altering immune responses, particularly at barrier sites such as the lung.^11^ Given the compositional similarities between the gut and lung microbiomes, recent studies have suggested potential parallels in the regulatory mechanisms of the gut–brain axis and lung–brain axis.^12^ Given that inhalation constitutes the primary route through which humans are exposed to environmental factors, the lung is likely to act as a key factor in the role of the lung–brain axis in epilepsy pathogenesis.^13^ The lung and brain maintain intricate immunological crosstalk that critically modulates disease progression across pathological contexts,^14^ forming the “lung–brain axis"—a biological cascade driven primarily by immune signaling. In common pulmonary disorders (*e.g.*, asthma, pneumonia, and chronic obstructive pulmonary disease), localized lung inflammation elicits proinflammatory cytokine release; these molecules disseminate systemically to induce neuroinflammation and disrupt blood–brain barrier integrity.^15^ Beyond soluble factors, activated immune cells in diseased lungs migrate to the brain, directly exacerbating nerve injury and reinforcing pulmonary–neurological pathological links.^16^ Supporting this, a recent bidirectional Mendelian randomization study using public datasets provided compelling evidence that COVID-19 may drive optic nerve and visual pathway diseases,^17^ validating the lung–brain axis as a key regulatory mechanism and highlighting its clinical relevance for addressing pulmonary infection-related neurological sequelae. Collectively, these findings suggest that the lung–brain axis acts as a pivotal interface: its dysfunction connects pulmonary and neurological pathology, but targeting it may be a novel therapeutic strategy for the treatment of lung disorders and their associated neural complications.

**Figure 1:**
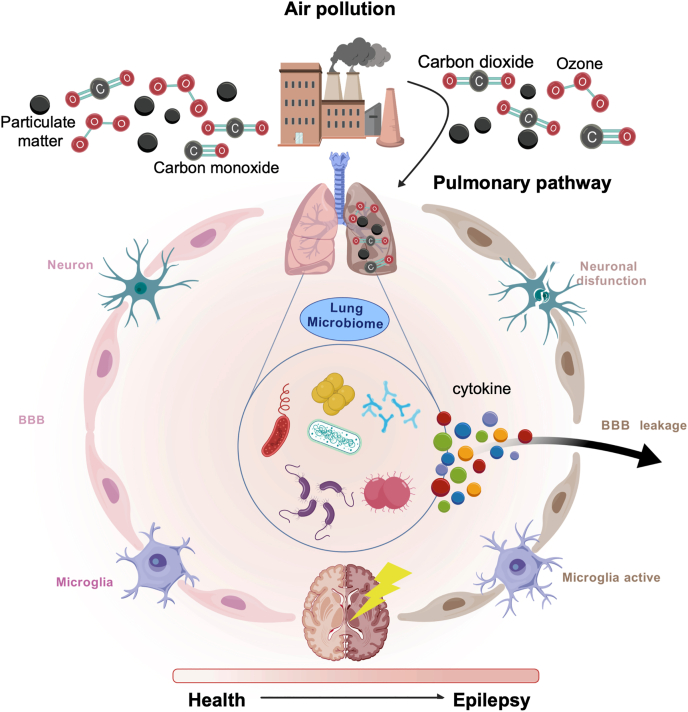
Air pollution inhalation potentially leads to increased susceptibility to epilepsy via the lung–brain axis. The figure was created on https://BioGDP.com.

This article provides a narrative review of the putative role of the lung–brain axis in the development of epilepsy (Fig. 1). Specifically, it focuses on three core areas: first, the epidemiological evidence linking environmental factors to epilepsy susceptibility, including associations between elevated air pollution levels and increased risks of new-onset clinical seizures as well as subclinical seizures; second, the potential mechanisms focusing on the lung–brain axis, encompassing immunological, neural (*e.g.*, vagal nerve signaling), neuroendocrine, and metabolic pathways that bridge pulmonary perturbations to epileptic pathogenesis; and third, the role of the lung microbiome, covering how environmental insults disrupt lung microbiota homeostasis, the interplay between lung microbiome dysbiosis and lung–brain axis activation, and the subsequent modulation of epilepsy development via microbial metabolites or immune crosstalk.

### Epidemiological evidence: Environmental factors and epilepsy susceptibility

The World Health Organization identifies air pollution as a primary public health risk, ranking it as the 12th leading global factor reducing disability-adjusted life years.^18^^,^^19^ By definition, air pollution is not a single entity but a complex mixture of diverse components, including particulate matter (PM), toxic gases, heavy metals, and organic compounds. These pollutants originate from a wide range of sources—outdoor sources such as industrial emissions, vehicle exhaust, and biomass combustion—as well as from indoor sources such as tobacco smoke, cooking fumes, and household fuel burning.^18^^,^^20^ These pollutants, acting alone or in combination, threaten human health. Their effects extend beyond the respiratory system to impact multiple organs, including the brain, and have been potentially linked to epilepsy.

### Evidence from human population studies and key exposures

While the role of air pollution in epilepsy remains an emerging field, several lines of evidence from human populations and pathological studies suggest a potential link. The primary focus here is on epidemiological and clinically relevant findings (Table 1).

**Table 1:** Summary of key studies on air pollution and neural outcomes relevant to epilepsy.

Study (author, year)	Study design	Exposure/intervention	Key findings related to epilepsy	Limitations
Choi et al, 2023^8^	Population-based retrospective cohort	Ambient air pollution (PM_10_, NO_2_, SO_2_, CO)	Higher pollutant levels were associated with an increased risk of neurological diseases, including epilepsy.	Exposure based on residential address (area-level); potential for unmeasured confounding.
Calderón-Garcidueñas et al, 2002^23^	Pathological case–control	Chronic exposure to severe urban air pollution	Exposed subjects showed brain inflammation, blood–brain barrier disruption, and neurodegenerative changes—hallmarks of a pro-epileptic environment.	Does not report direct epilepsy incidence; focuses on neuropathology.
Mallok et al, 2015^26^	Preclinical intervention	Acute ozone (O_3_) exposure	Ozone exposure reduced seizure severity and mortality in the PTZ model.	Acute exposure in a chemical seizure model; relevance to chronic human epilepsy is unclear.
Pauletti et al, 2019^25^	Evidence synthesis	Particulate matter	Summarizes evidence that particulate matter induces oxidative stress and neuroinflammation, key factors in epileptogenesis.	Does not present new primary epidemiological data.

PM, a prevalent and hazardous air contaminant,^21^ is categorized by aerodynamic diameter (*e.g.*, PM2.5, PM10), with smaller particles posing potentially greater risks due to deeper lung penetration.^22^

Evidence indicates that air pollutants target epileptogenesis or seizure-related processes via pathological interactions; as Calderón-Garcidueñas et al^23^ emphasized, air pollution-induced brain damage disrupts neural structure and function—with CNS changes (elevated excitability and cellular death) aligned with seizure-associated neural instability—which may increase the risk of epilepsy.^24^ Pauletti et al^25^ reported that PM exposure may induce oxidative stress, neuroinflammation, and brain cell death—key factors linked to epileptogenesis. These findings align with those of the air pollution-related neurotoxicity framework of Calderón-Garcidueñas et al,^23^ which emphasizes that pollutants trigger cascades that disrupt brain homeostasis, thereby forming a pathological environment conducive to neurological disorders (including potential epilepsy). Vulnerable populations, including elderly people and children, may suffer more severe impacts because of relatively weaker CNS defense mechanisms.

Concerning gaseous pollutants such as ozone, the evidence presents a more complex picture. Mallok et al^26^ demonstrated that ozone exposure protects against pentylenetetrazol-induced seizures in mice. This protective effect observed under specific experimental conditions (acute ozone exposure in a chemical seizure model) may reflect a hormetic or biphasic response to oxidative stress, where low-level stressors can induce adaptive cellular defenses. Importantly, this contrasts with the chronic, low-grade neuroinflammation and neural damage widely documented in epidemiological and pathological studies of long-term exposure to complex air pollution mixtures, which is the primary focus of our hypothesis.

### Critical appraisal and limitations of current evidence

While air pollution has demonstrable neurotoxic effects in humans and experimental models, its specific role in epilepsy requires further elucidation. The current epidemiological evidence, while suggestive, has notable limitations. These include the relative scarcity of large-scale studies designed specifically to assess epilepsy incidence, the challenge of precise individual-level exposure assessment often relying on area-level estimates, and the difficulty in fully accounting for potential confounding factors such as socioeconomic status. Furthermore, the apparent discrepancy between some experimental findings (*e.g.*, acute ozone effects) and observations from chronic human exposure studies underscores the importance of exposure context. Despite these limitations, the consistency of pathological findings linking PM exposure to a pro-epileptogenic brain environment provides a compelling basis for the hypothesized association.

### Biological plausibility: mechanistic links via the lung–brain axis

The epidemiological and pathological associations described above are supported by research into potential mechanisms, primarily involving the lung–brain axis. Upon inhalation, PM2.5 induces the release of circulating proinflammatory cytokines (*e.g.*, TNF-α and IL-6), which activate Toll-like receptors (particularly TLR4) in brain endothelial cells, triggering downstream inflammatory signaling, promoting additional proinflammatory mediators (*e.g.*, IL-1β), and disrupting blood–brain barrier integrity—conditions that may drive neuroinflammation and cognitive dysfunction.^22^ Studies have shown that the lung–brain axis is a critical mediator that regulates the proinflammatory environment in the brain induced by inhaled pollutants. Specifically, Mumaw et al demonstrated that the inhalation of gaseous pollutants such as ozone (O3), which cannot directly penetrate the brain, triggers the release of circulating signaling factors from the lungs to the brain; these factors then transmit systemic signals to activate neurotoxic pathways, thereby exacerbating neurotoxicity.^27^ In addition to gaseous pollutants, PM such as PM2.5 exerts neurological effects through pathways linked to the lung–brain axis. Its primary route to the brain is via the airways, where pulmonary exposure initiates a cascade of responses. Studies suggest that PM2.5 may increase blood‒brain barrier permeability; this disruption allows the entry of PM2.5 into the brain and triggers the production of brain-targeted autoantibodies, potentially altering the pathophysiology of neurological disorders.^28^, ^29^ In support of these findings, Diamond et al reported that circulating autoantibodies can infiltrate the brain through a compromised blood–brain barrier to directly contribute to the pathogenesis of neurological illness, revealing another critical mechanism through which the lung–brain axis can mediate pollutant-induced brain harm, as pulmonary-derived signals such as autoantibodies access the brain via disruption of the blood–brain barrier.^29^ Additionally, the lung–brain axis may modulate neurological function by regulating inflammatory signaling. Inhaled pollutants induce pulmonary inflammation, which stimulates the release of proinflammatory cytokines that travel to the brain via systemic circulation—a process orchestrated by the lung–brain axis. For instance, Vezzani and Baram reported that IL-1β, a key inflammatory cytokine, plays a pivotal role in epileptogenesis by inducing neuronal hyperexcitability and excitotoxicity.^30^ These findings suggest that pollutant-induced activation of the lung–brain axis may activate IL-1β signaling to promote neurological dysfunction.

## Potential mechanisms through which the lung–brain axis contributes to epilepsy pathogenesis

**Figure 2:**
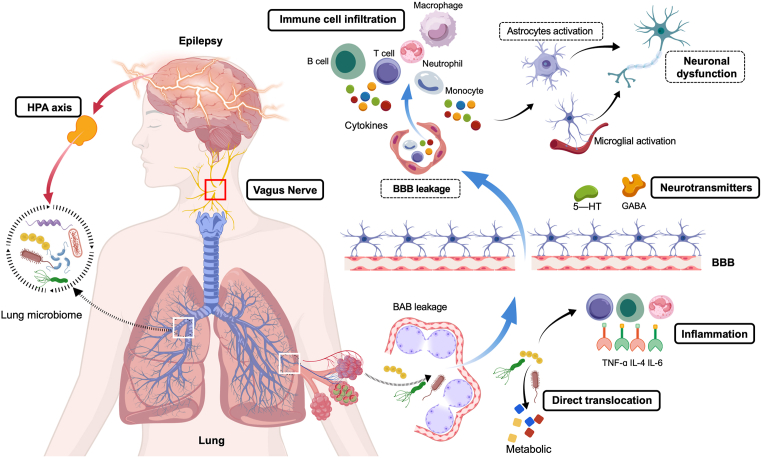
Potential channels of communication via the lung–brain axis between the lung microbiota and epilepsy. The vagus nerve, the HPA axis, the direct translocation of microorganisms, the effects of lung microbes on neuroimmune and inflammatory pathways, and metabolic alterations that may penetrate the blood‒brain barrier to disrupt immune cells and neurons in the brain all contribute to the communication between the lung and the brain and affect epilepsy pathogenesis. CNS, central nervous system; BBB, blood–brain barrier; BAB, blood–air barrier; HPA, hypothalamus–pituitary–adrenal. The figure was created on https://BioGDP.com.

The lung undergoes physiological changes driven by the interplay between microbial endocrinology and environmental factors.^31^ The role of the lung microbiota in the regulation of the CNS has attracted increasing attention (Table 2). Based on existing evidence, we propose five mechanisms underlying the regulation of epilepsy via the lung–brain axis: i) direct translocation of microorganisms; ii) disruption of neuroimmune/inflammatory pathways by lung bacteria; iii) vagus nerve-mediated transmission of proseizure signals from the lungs to the brain; iv) hypothalamus–pituitary–adrenal axis perturbations; and v) metabolic shifts (Fig. 2). These mechanisms underscore that the lung–brain axis is a novel pathway involved in epilepsy pathogenesis.

**Table 2:** Selected studies supporting mechanistic links between the lung–brain axis and epilepsy pathophysiology.

Study (author, year)	Primary disease/experimental model	Key finding related to the lung–brain axis	Implications for epilepsy/mechanistic relevance
Hosang et al, 2022^57^	Rat model of multiple sclerosis: Experimental autoimmune encephalomyelitis	Alterations in the lung microbiome (*e.g.*, via antibiotic treatment or microbial exposure) directly modulated the induction and severity of brain autoimmunity, influencing central nervous system-infiltrating T cells.	Provides direct experimental proof that lung-derived microbial signals can regulate adaptive immune cell trafficking into the central nervous system and the severity of neuroinflammation—a key pathway in immune-mediated epileptogenesis.
Villalba et al, 2023^32^	*Pseudomonas aeruginosa* lung infection (mouse)	Lung infection induced anxiety-like behavior, hyperlocomotion, neuroinflammation (microglial activation), and blood–brain barrier dysfunction.	Demonstrates a direct link from bacterial lung infection to behavioral changes, blood–brain barrier disruption, and neuroinflammation, establishing a causal pathway relevant to infection-associated epilepsy.
Mumaw et al, 2016^27^	Ozone (O_3_) exposure (mouse)	Inhalation of ozone triggered the release of circulating factors from the lung that primed microglia in the brain towards a pro-inflammatory phenotype.	Identifies a humoral signaling pathway (lung→circulation→brain) by which a gaseous pollutant primes brain immune cells (microglia), linking environmental exposure to a pro-inflammatory central nervous system state conducive to seizures.
Bryche et al, 2020^41^	Respiratory syncytial virus (RSV) infection (mouse)	RSV, a pulmonary pathogen, demonstrated tropism for olfactory sensory neurons, suggesting a potential neural route for central nervous system invasion.	Illustrates a potential direct neural pathway (via cranial nerves) for a respiratory virus to reach the brain, relevant to understanding viral infection-triggered seizures and encephalitis.
Li et al, 2020^53^	PM2.5 exposure (mouse)	Inhalation of PM2.5 altered the composition and metabolic profile of the lung microbiome and induced pulmonary inflammation.	Establishes that a major environmental pollutant directly perturbs lung microbiota homeostasis and triggers local inflammation, the proposed initial step in the pollutant→lung dysbiosis→brain cascade relevant to epilepsy.
Calderón-Garcidueñas et al, 2002/2015^23^^,^^28^	Chronic urban air pollution exposure (human, children/young adults)	Documented significant neuroinflammation, blood–brain barrier breakdown, and neurodegenerative pathology in the brains of individuals exposed to severe air pollution.	Provides critical human pathological evidence that chronic pollutant exposure creates a pro-epileptogenic brain environment characterized by inflammation, excitability, and neuronal damage, bridging epidemiology and mechanism.

### Direct translocation

The first proposed mechanism involves the direct translocation of microorganisms or their products across the alveolar‒capillary barrier. Once these microorganisms or their byproducts enter the bloodstream, they may reach the CNS and potentially influence epileptic seizures. Additionally, mice with lung infections exhibit anxiety-like behaviors and hyperlocomotion, which suggests that pathological changes in the lungs can affect neurological function.^32^ Recent mouse model research has demonstrated that the introduction of lipopolysaccharide into the lungs leads to lung microbial dysbiosis, which alters the translocation of the microbiota into the bloodstream.^31^ These findings indicate that the lung microbiota or its components can enter the circulatory system through direct translocation, providing a potential pathway to impact the CNS. However, direct experimental evidence for this pathway is currently lacking. Specifically, it remains unknown whether lung microbiota-derived components (*e.g.*, lipopolysaccharide or metabolites) translocate to the brain under non-infectious, pollution-like conditions, and subsequently lower seizure thresholds in established epilepsy models. Future studies employing labeled microbial products and electrophysiological monitoring in seizure models are needed to validate this pathway.

Nerve cells are susceptible to viral infections, and neurotropic viruses that infect nerve cells can cause neurological disorders via blood circulation, peripheral routes, or cranial nerve axons.^33^ Human metapneumovirus (HMPV), a common respiratory pathogen, can establish persistent infection in the lungs and may affect neurological function via chronic immune modulation or indirect pathways.^33^ These findings suggest that respiratory viruses can translocate and exert long-term neurological effects. Collectively, these findings suggest that the lung microbiota and its products, as well as respiratory viruses, may influence epileptic seizures through direct translocation across the alveolar‒capillary barrier, entry into the bloodstream, and subsequent penetration of the blood–brain barrier or invasion via neural pathways.

### Neuroimmune/inflammatory pathways

Epilepsy is strongly associated with neuroimmunity and neuroinflammation. Peripulmonary bacteria and lung microbial communities stimulate proinflammatory mediator production, which modulates systemic humoral components; these mediators, together with host antimicrobial peptides, regulate humoral immunity that extends to the CNS through systemic communication. Systemic humoral factors such as endogenously secreted antibodies also complement protein-mediated immune responses, forming a network that links pulmonary immune activity to CNS function.^33^ It is established that the lung microbiota affects systemic cell-mediated immunity to further impact CNS physiology.

In the CNS, microglia and astrocytes are highly prone to inflammatory activation, and their proinflammatory state is a key driver of epileptogenesis.^34^ Studies on germ-free and antibiotic-treated animals have shown altered microglial morphology and defects in maturation, activation, and differentiation, leading to inadequate immune responses against pathogens; importantly, gut microbiome colonization reverses these deficits, indicating that microbial diversity—including that of the lung microbiota—is essential for proper microglial function and directly relevant to epilepsy pathogenesis.^35^ In addition to resident glial cells, peripheral immune cells such as T cells and monocytes (which infiltrate brain tissue) are linked to epilepsy morbidity. Lung microbiota-induced disruption of systemic immune homeostasis may perturb CNS immunity; in contrast to the historical view of the CNS as an immune-privileged site, recent research confirms that it is an actively regulated immune surveillance site, allowing peripheral immune signals to influence its environment.^36^ For example, monocytes can differentiate into macrophages, infiltrate the brain, and transform into “microglia-like cells” that exacerbate epileptic symptoms.^37^ Pulmonary immune activation breaches the blood–brain or blood–cerebrospinal fluid barrier and enables the infiltration of immune cells and microbiota-derived substances, directly triggering seizures.^37^ The high immune cell density in the lung suggests that it is a key player in lung–brain axis regulation in epilepsy; however, specific mechanisms and direct lung epilepsy research remain limited. While pulmonary inflammation can induce neuroinflammation, a direct causal link between lung microbiota-driven, pollutant-induced neuroinflammation and the initiation or exacerbation of epileptic seizures remains to be experimentally established. Studies correlating specific lung microbial shifts with brain cytokine profiles and seizure susceptibility in animal models are warranted. Nevertheless, current evidence strongly supports the hypothesis that the lung microbiota induces immune and inflammatory responses via the lung–brain axis, contributing to epileptic seizure development.

### Nervous system

The lungs and airways are predominantly innervated by nerve fibers from vagal sensory neurons, establishing a direct neural connection between the pulmonary system and the CNS that serves as a key anatomical basis for the lung–brain axis.^38^ These vagal sensory terminals play a pivotal role in the pulmonary parasympathetic inflammatory response: upon stimulation, they secrete neuropeptides or acetylcholine to regulate pulmonary function and transmit signals to the brain, bridging lung activity and CNS physiology.^39^ This makes the vagus nerve a potential hub for interactions between the lung microbiota and neural signaling, as lung microbial components or metabolites may stimulate vagal terminals to trigger brain-targeted neural responses. Supporting this view, vagus nerve stimulation is an approved epilepsy therapy, underscoring its role in modulating epileptic activity.^40^ Given its dual involvement in pulmonary function regulation and CNS modulation, the vagus nerve plausibly mediates interactions between the lung microbiota and epileptic seizures.

Beyond direct vagal signaling, neural pathways facilitate pulmonary influences on the CNS via infectious processes. For example, anterograde transport through olfactory neurons is a potential route for respiratory syncytial virus—a pulmonary pathogen—to reach the CNS, although further characterization of its nasal tropism is needed.^41^ Similarly, SARS-CoV-2 infection is linked to neurological symptoms, with the virus spreading from peripheral neurons to the CNS via anterograde or retrograde transport.^42^ These findings suggest that pulmonary infections—possibly influenced by the lung microbiota—induce neural remodeling in the lungs and brain, potentially altering neural excitability to contribute to epileptic activity.

Taken together, these findings suggest that the vagus nerve—via pulmonary sensory innervation, inflammatory signaling modulation, and established epilepsy relevance—emerges as a central conduit in the lung–brain axis, likely mediating the influence of the lung microbiota on epileptic seizures through neural pathways.

### Hypothalamus‒pituitary‒adrenal axis

The hypothalamic–pituitary–adrenal axis, a key neuroendocrine pathway, centralizes stress response regulation by controlling the production of corticotropin-releasing factor, adrenocorticotropic hormone, and downstream catecholamines and glucocorticoids.^43^ It is intimately linked to epilepsy: epileptic patients have elevated glucocorticoid levels, and the stress-activated hypothalamic–pituitary–adrenal axis exacerbates epilepsy via enhanced glutamatergic signaling and seizure induction.^44^ Intriguingly, its derived hormones exert divergent seizure effects—corticotropin-releasing factor and corticosterone induce seizures, while most deoxycorticosteroids act as anticonvulsants.^44^ Beyond stress and seizure modulation, the hypothalamic–pituitary–adrenal axis plays a role in pulmonary physiology. For example, melanocortins—critical for their function in embryonic and fetal development—also regulate lung development, bridging neuroendocrine and pulmonary biology.^45^ Additionally, it modulates immune and inflammatory responses via endocrine signaling and peripheral nervous system regulation; under stress, it drives the production of cortisol (an anti-inflammatory mediator) and hypothalamic corticotropin-releasing factor to maintain systemic immune homeostasis.

These connections suggest a plausible lung–brain axis pathway: the lung microbiota may influence hypothalamic–pituitary–adrenal axis activity by altering stress-induced hormone secretion, modulating pulmonary–neuroendocrine crosstalk, or affecting the regulation of immune and inflammatory responses, thereby affecting epileptic susceptibility.^46^ However, the specific links between the lung microbiota, the hypothalamic–pituitary–adrenal axis, and epilepsy remain underexplored and require further investigation. It is important to note that while stress and hypothalamus–pituitary–adrenal axis dysfunction are implicated in epilepsy, and the gut microbiota is known to influence hypothalamus–pituitary–adrenal axis activity, direct evidence linking lung microbiota perturbations to hypothalamus–pituitary–adrenal axis alterations in epilepsy is scarce. Future studies should investigate whether specific lung microbial profiles correlate with cortisol levels or stress responsiveness in epileptic patients or relevant animal models.

### Neurotransmitters

Neurotransmitter imbalance is closely linked to epilepsy, with epileptic foci characterized by decreased γ-aminobutyric acid, increased glutamate, elevated dopamine and norepinephrine, and reduced serotonin levels.^47^ Patients with temporal lobe epilepsy exhibit serotonin deficiency, highlighting neurotransmitter dysregulation in seizure pathogenesis.^47^ These findings suggest that epilepsy may be associated with lung-derived neurotransmitter changes mediated by the lung–brain axis. Emerging microbial research—primarily on the gut microbiota—supports this link and provides a framework for understanding lung microbiota effects. For example, *Akkermansia muciniphila* and *Parabacteroides distasonis* colonization alters serum amino acid levels, which modify hippocampal concentrations of seizure-related glutamate and γ-aminobutyric acid to confer protective antiseizure effects in mice.^48^ Extending this logic, lung microbiota-derived metabolites may similarly influence systemic amino acid pools or regulate neurotransmitter synthesis, impacting brain glutamate and γ-aminobutyric acid levels. Other epilepsy-related neurotransmitters also have lung–brain cross-talk potential. Norepinephrine has dose-dependent effects on epileptogenesis, with its levels potentially modulated by pulmonary microbial activity.^47^ Additionally, dopamine, serotonin, and acetylcholine—all strongly linked to epilepsy—regulate peripheral receptors and interact with the vagus and enteric nervous systems, creating indirect pathways for peripheral (including pulmonary) neurotransmitter changes to influence brain function.^49^ The hypothesis that lung microbiota-derived metabolites influence brain neurotransmitter levels is primarily extrapolated from gut microbiome research. Direct measurement of changes in lung-derived microbial metabolites (*e.g.*, short-chain fatty acids, though less abundant than in the gut) in systemic circulation or their impact on central glutamate/γ-aminobutyric acid balance following pollutant exposure remains an important area for future investigation. Studies employing metabolomic profiling coupled with seizure monitoring in models of lung dysbiosis are warranted to establish causality.

Taken together, these findings suggest that lung microbiota metabolites may shape neurotransmitter dynamics via systemic metabolic effects or lung–brain axis neural signaling, thereby contributing to epileptic susceptibility. Further research is needed to clarify how lung-specific microbial activity directly modulates seizure-relevant neurotransmitter pathways.

## Role of the lung microbiome in epilepsy

### Impact of environmental insults on lung microbiota homeostasis

The microbiota is defined as “the ecological community of commensal, symbiotic, and pathogenic microorganisms that share our body space”.^50^ The lung and lower respiratory tract microbiota contain bacteria that are also prevalent in the upper respiratory tract. Healthy lungs typically harbor the phyla Firmicutes, Bacteroidetes, Proteobacteria, Fusobacteria, and Actinobacteria, with distinct operational taxonomic units, including Streptococcus species, Veillonella, and Prevotella.^51^ The lung microbiota is characterized by its dynamic composition, influenced by continuous microbial inhalation, mucociliary clearance, and local immune activity. It is distinct from the upper respiratory tract microbiota; while sharing some taxa, the healthy lung maintains a unique community shaped by the alveolar microenvironment.

Characterizing the pulmonary microbiome is critical for evaluating the effects of environmental factors on lung and brain health. The respiratory tract extends from the oral cavity to the trachea and lungs, and while the lung was once considered sterile, emerging research has shown that manipulating the pulmonary microbiota activates immune cells in the brain and influences neurological disease development.^52^ Its low biomass, compared with the gut, makes it particularly susceptible to perturbation by external factors like pollutants. For example, exposure to environmental pollutants such as PM2.5 significantly alters the composition of the pulmonary microbiome. A recent study has demonstrated that PM2.5 inhalation induces acute bronchial inflammation, increases goblet cell counts, and reduces the abundance of Proteobacteria in the lung,^53^ confirming that environmental factors disrupt the pulmonary microbiota balance.

### The interplay between lung microbiome dysbiosis and lung–brain axis activation

Evidence suggests that the lung–brain axis is related to epilepsy. For example, up to 39% of patients with neurological sequelae from acute respiratory syncytial virus present with seizures, status epilepticus, central apnea, and encephalitis.^41^ Potential pathogenic microorganisms—linked to elevated inflammatory markers in stable illness—produce increased levels of proinflammatory cytokines that impact microglial activation, the neuroimmune microenvironment, and lung inflammation regulation, thereby contributing to neurological disorders through multiple pathways.^10^ Given that the pulmonary microbiota influences proinflammatory cytokine production and is affected by environmental factors, it is reasonable to hypothesize that environmental factors may impact epilepsy via the lung–brain axis by altering the pulmonary microbiota, although further research is needed to confirm this connection.

The underlying causes of many neurological disorders remain poorly understood, but growing evidence highlights the role of the microbiome—especially its role in cross-organ communication—as a key research area. While the gut–brain axis has long been the primary focus for understanding microbiome-mediated CNS regulation, emerging research underscores the significance of other microbial niches (including the lung) in influencing neurological function. For example, in a mouse model of pentylenetetrazol-induced seizures, intestinal inflammation exacerbated convulsive activity and reduced antiepileptic drug efficacy, while alleviating intestinal inflammation resulted in antiepileptic benefits.^54^ This finding points to a broader pattern: microbial dysbiosis across multiple organs shares commonalities that may drive neurological dysfunction.

Such cross-organ relationships extend beyond the gut. Bell et al reported that pathways linking the microbiome to neurological disorders spanned from the nose to the gut, encompassing CNS-specific immunity and direct transfer of microorganisms or their metabolites.^31^ Importantly, the gut and lung microbiota are interconnected—modifying the host diet indirectly alters the lung microbial composition and can promote a healthy gut microbiome, potentially amplifying neurological effects.^55^ This gut–lung–microbiome crosstalk suggests that microbial signals from one mucosal surface can influence distant organs such as the brain.

Within this framework, the lung microbiota—comprising bacteria, viruses, and fungi^9^^,^^56^—emerges as a critical player in lung–brain axis communication. Although the lung has a low microbial load, Hosang et al demonstrated a robust link between lung microbiome alterations and CNS inflammation, showing that lung microbial shifts modulate CNS autoimmune disorder risk.^57^ Given this phylogenetic overlap, the lung and gut microbiota share dominant phyla (Firmicutes and Bacteroidetes), suggesting the presence of conserved mechanisms through which microbes influence systemic and neural responses.^57^

### Microbial mediators and the modulation of epileptogenesis

One potential mechanism involves the microbial production of neurochemicals such as γ-aminobutyric acid, which may directly affect neuronal excitability (a key epileptogenesis factor); however, research on lung microbiota-derived neurochemicals is nascent and parallels well-studied gut microbiota–diet interactions, offering a promising direction. Additionally, the role of the microbiome in maintaining immune homeostasis is pivotal, and microbial signaling across mucosal surfaces impacts inflammation and neurodegeneration, two hallmarks of CNS disorders.^31^

Clinical observations further support these links. For example, neurological sequelae in COVID-19 patients (including stroke and poststroke epilepsy) highlight how pulmonary insults propagate to the brain, although the specific pathophysiological role of the lung microbiota in such patients needs further investigation.^58^

To delineate the unique potential of the lung microbiota in epilepsy, a direct comparison with the gut microbiota is instructive. In contrast to the dense, stable, and nutrient-rich gut ecosystem, the lung harbors a low-biomass, dynamic microbiome, perpetually remodeled by inhalation, mucociliary clearance, and local immune surveillance. This ecological niche renders it exquisitely susceptible to disruption by airborne pollutants. Furthermore, their primary communication pathways with the brain differ substantially: while the gut–brain axis predominantly utilizes humoral routes (*e.g.*, microbial metabolites entering portal circulation), the lung is densely innervated by the vagus nerve, providing a direct neural conduit for rapid signal relay to epilepsy-relevant brainstem and limbic regions. Phylogenetically, the lung microbiome is also distinct, being seeded and influenced more by the upper respiratory tract than by the gastrointestinal tract. Collectively, these attributes position the lungs not as a mere “second gut” but as a first-line, neural-integrated environmental sensor. Its dysbiosis may thus modulate brain excitability through mechanisms that are both complementary to and distinct from those of the gut–brain axis.

Collectively, these findings suggest a compelling case in which the lung microbiota may significantly influence epilepsy via the lung–brain axis, with mechanisms involving immune modulation, neurochemical signaling, and cross-organ microbial crosstalk. Clarifying these pathways as research advances could provide more evidence supporting the lung microbiome as a key player in epileptogenesis.

## Conclusions and perspectives

Emerging evidence highlights a strong link between epilepsy and environmental exposures—especially air pollution—with the lungs acting as the primary interface. Inhaled pollutants disrupt pulmonary immune and neuronal microenvironments, triggering cascades that propagate to the brain via the lung–brain axis. Parallel to the well-characterized gut–brain axis, the lung–brain axis—shaped by lung microbiota dynamics—has emerged as a critical yet underexplored pathway in epileptogenesis. The role of the lung as a gateway for environmental and microbial signals, combined with its dense neural and immune connections to the CNS, positions it as a key mediator of systemic influences on brain excitability and susceptibility to seizures.

This review synthesizes the hypothesis that the lung–brain axis contributes to epilepsy pathophysiology via interconnected mechanisms: immune/inflammatory signaling (through cytokines, microbial metabolites, and peripheral immune cell infiltration), neural communication (mainly via the vagus nerve), neuroendocrine regulation (via hypothalamus–pituitary–adrenal axis modulation), and neurotransmitter metabolism (*e.g.*, glutamate, γ-aminobutyric acid, and catecholamine balance). These pathways redefine the lung not only as a respiratory organ but also as a sensory and regulatory hub that shapes CNS function—including the hyperexcitability underlying seizures.

Critical gaps persist. Future preclinical research must establish causality by investigating, for example, whether lung microbiota dysbiosis directly lowers seizure thresholds or whether pulmonary inflammation exacerbates epileptic activity via specific molecular mediators. Clinical research—including longitudinal cohort studies and microbiota profiling in epileptic patients with comorbid lung conditions—is essential for the translation of animal findings to human pathology. Additionally, isolating the unique contributions of the lung–brain axis from those of other systemic pathways requires targeted interventions.

**Figure 3:**
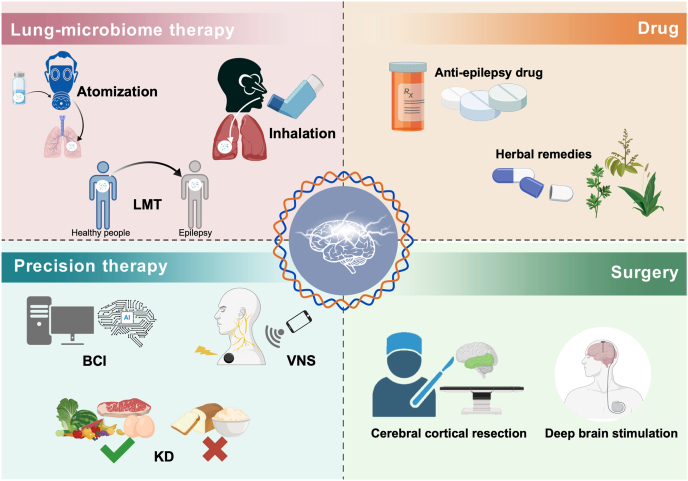
Potential interventions in the lung microbiota for mitigating the progression of epilepsy. On the basis of current evidence, interventions such as lung microbiome-based therapies (atomization, inhalation, and LMT) that target the lung hold promise as potential strategies for preventing and treating the progression of epilepsy. Other types of treatments include drugs (antiseizure medications, herbal remedies), surgery (cerebral cortical resection, deep brain stimulation), and precision therapy (BCI, VNS, KD). LMT, lung microbiota transplantation; BCI, brain–machine interface; VNS, vagus nerve stimulation; KD, ketogenic diet. The figure was created on https://BioGDP.com.

Beyond elucidating novel pathophysiological mechanisms, the lung–brain axis hypothesis carries immediate and actionable public health implications. Foremost among these is the reinforcement of air quality improvement as a critical, population-wide preventive measure against neurological disorders, including epilepsy (Fig. 3). Prevention strategies—our synthesis suggests that reducing the inhalation of PM and gaseous pollutants or restoring lung microbiota homeostasis via probiotics could mitigate the dysbiosis of the lung microbiome and the subsequent neuroinflammatory cascade via the lung–brain axis, thereby potentially reducing the risk of epileptogenesis and seizure exacerbation in susceptible individuals. Treatment advances may involve identifying key mediators along the axis to develop novel therapies, such as inhaled anti-inflammatory agents to dampen neuroinflammatory cascades, vagus nerve modulation to normalize neural signaling, or precision microbiota-based interventions to rebalance pulmonary–CNS communication. More immediately testable strategies can be extrapolated from adjacent fields. Of note, oral and inhaled probiotics/prebiotics are under investigation for respiratory conditions such as asthma and chronic obstructive pulmonary disease. Their documented potential to modulate local and systemic immune-inflammatory responses provides a relevant preclinical rationale for evaluating their impact on seizure susceptibility in models where lung inflammation and the lung–brain axis are implicated. In contrast, highly targeted approaches such as precise lung microbiota transplantation or inhaled neuro-modulatory agents remain fundamentally conceptual. Their future feasibility is contingent upon prior identification of specific, causally implicated microbial consortia or signaling molecules within the lung–brain axis from mechanistic studies. Therefore, therapeutic translation logically follows mechanistic discovery. Prioritizing research to establish causal links between lung dysbiosis, axis activation, and epileptogenesis is the critical first step. This foundational knowledge will then rationally prioritize the most promising intervention points for development. For epilepsy, a disorder where a significant proportion of patients experience uncontrolled seizures despite available medications, targeting modifiable environmental risk factors represents a complementary and essential strategy alongside pharmaceutical development. Public health policies aimed at curbing air pollution thus emerge as a form of non-pharmacological, primary prevention for epilepsy, potentially alleviating the global burden of this disease.

In summary, the lung–brain axis represents a paradigm shift in epilepsy research, moving beyond CNS-centric models to embrace the integration of the brain with peripheral organs and the environment. Revealing its complexities will deepen the understanding of the multifactorial origins of epilepsy and enable the development of innovative, personalized prevention and treatment approaches—ultimately improving outcomes for millions of people with this debilitating condition. Interdisciplinary collaboration across neuroscience, pulmonology, immunology, and microbiology is urgently needed to fully realize the potential of targeting the lung–brain axis in transforming epilepsy care.

## CRediT authorship contribution statement

**Zijun Lin:** Writing – review & editing, Writing – original draft, Visualization, Validation, Supervision, Software, Resources, Project administration, Methodology, Investigation, Formal analysis, Data curation, Conceptualization. **Di Zhou:** Resources, Investigation, Conceptualization. **Jialing Jiang:** Methodology, Conceptualization. **Patrick Kwan:** Writing – review & editing, Writing – original draft, Visualization, Validation, Supervision, Software, Resources, Project administration, Methodology, Investigation, Funding acquisition, Formal analysis, Data curation, Conceptualization. **Xin Tian:** Writing – review & editing, Writing – original draft, Visualization, Validation, Supervision, Software, Resources, Project administration, Methodology, Investigation, Funding acquisition, Formal analysis, Data curation, Conceptualization.

## Declaration of Competing Interests

Xin Tian is a member of the *Genes & Diseases* Editorial Board. To minimize bias, he was excluded from all editorial decision-making related to the acceptance of this article for publication. The remaining authors declare no conflict of interests.
